# Squamous Cell Carcinoma of the Skin in a Teenager with Fanconi Anemia: A Challenging Treatment

**DOI:** 10.3390/ijms27104366

**Published:** 2026-05-14

**Authors:** Ekaterina Zelenova, Tatiana Belysheva, Kristina Orlova, Vasily Grigorenko, Vera Semenova, Elena Sharapova, Yana Vishnevskaya, Igor Samoylenko, Tatiana Nasedkina, Timur Valiev, Vladimir Polyakov, Svetlana Varfolomeeva

**Affiliations:** 1 N.N. Blokhin National Medical Research Center of Oncology, Ministry of Health of the Russian Federation, 115478 Moscow, Russia; klinderma@bk.ru (T.B.); krisman03@gmail.com (K.O.); oncogrigorenko@yandex.ru (V.G.); sulpiridum@yandex.ru (V.S.); sharapovae.v@yandex.ru (E.S.); yana_vishn@list.ru (Y.V.); igor.samoylenko.uz@gmail.com (I.S.); timurvaliev@mail.ru (T.V.); vgp-04@mail.ru (V.P.); s.varfolomeeva@ronc.ru (S.V.); 2 Engelhardt Institute of Molecular Biology, Russian Academy of Sciences, 119991 Moscow, Russia; tanased06@rambler.ru; 3 Dmitry Rogachev National Medical Research Center of Pediatric Hematology, Oncology and Immunology, 117198 Moscow, Russia; 4 Department of Oncology, Sechenov First Moscow State Medical University, 119048 Moscow, Russia

**Keywords:** Fanconi anemia, *FANCA*, squamous cell carcinoma, immunotherapy, pediatric oncology, HSCT, pembrolizumab

## Abstract

Fanconi anemia (FA) is a rare inherited disorder associated with impaired DNA repair, characterized by congenital anomalies, bone marrow failure, and a significantly increased risk of developing malignancies, particularly squamous cell carcinoma (SCC) of the head and neck. Treatment options for advanced SCC in FA are limited due to hypersensitivity to DNA-damaging agents. This article presents a unique case of SCC that developed in a 17-year-old patient with FA caused by a homozygous mutation in the *FANCA* gene. At the age of 10, he received a bone marrow transplant from a compatible related donor. Conditioning therapy included busulfan, thymoglobulin, and fludarabine, while graft-versus-host disease (GvHD) prophylaxis was administered with rituximab, methotrexate, and cyclosporine A. Nevertheless, he developed chronic cutaneous GVHD, which was treated for four years with ruxolitinib and tacrolimus, achieving only partial control. During this period, locally advanced cutaneous SCC (T3N0M0, stage III) manifested on the face. Surgery, radiation therapy, and immunotherapy with pembrolizumab led only to an initial partial response. This first pediatric case of immunotherapy for SCC in FA highlights the challenges of treating this rare patient group. Nevertheless, combining radiation therapy with immunotherapy may represent a possible option for disease control.

## 1. Introduction and Clinical Significance

Fanconi anemia (FA) is a rare, mainly autosomal recessive disease associated with multiple malformations, bone marrow failure, and a high risk of malignant neoplasms [1,2]. X-linked recessive (*FANCB*) and autosomal dominant (*FANCR*/*RAD51*) types of inheritance are much less common.

Currently, 23 genes associated with FA have been identified [3,4]. Pathogenic variants in the FANC family genes lead to the inactivation of DNA repair. This is associated with a high risk of large chromosomal aberrations, which can be confirmed by a test with diepoxybutane (DEB) or mitomycin C which is routinely used [5].

One of the life-threatening complications—bone marrow failure—can be detected from the age of 7.6 years, and 90% of patients have signs of bone marrow failure by the age of 40 [6]. Hopefully, hematopoietic stem cell transplantation (HSCT) can successfully cure this condition [7]. Nevertheless, patients also have an age-dependent risk of neoplasm—leukemia and embryonic tumors (nephroblastoma, neuroblastoma, and medulloblastoma) predominate in childhood [3,8], while head and neck cancer is more common in the older age group [9].

The incidence of squamous cell carcinoma (SCC) of the head and neck in patients with FA is more than 500 times higher compared to the general population. The mean age of manifestation of SCC is 31 years (range 15–49 years), the overall survival rate is 17 months, and the 5-year survival rate is 39%. In half of the cases, local relapse is observed, and second tumors are seen in more than 60% of patients [9]. In pediatric patients, SCC is a rare malignancy, and only isolated cases have been reported in children aged 11 years or older [10,11]. The oral cavity is the most common site of SCC manifestation with approximately 60% of FA-associated carcinomas affecting the oral tongue, while skin lesions were described less commonly [12].

Here, we present the first pediatric case of locally advanced cutaneous facial SCC in a patient with FA following HSCT, who was treated with multimodal therapy including pembrolizumab. Our experience highlights the limited efficacy and significant toxicity of immunotherapy in this unique setting, underscoring the urgent need for caution and alternative strategies.

## 2. Case Presentation

### 2.1. Patient History

**Figure 1:**
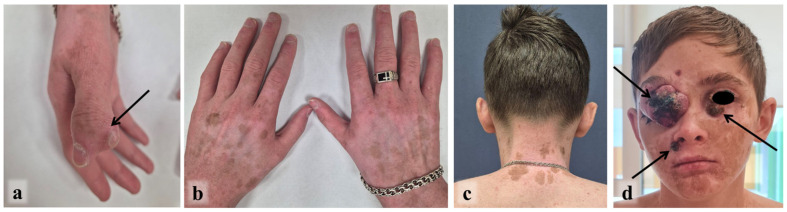
Examination of the patient (September 2024, 16 y.o.): the accessory nail phalanx of the thumb of the right hand, indicated by an arrow (**a**), chronic GVHD after allogeneic HSCT (**b**–**d**) and locally advanced SCC of the facial skin, indicated by arrows (**d**).

A 17-year-old male, the third child of non-consanguineous parents, was diagnosed with Fanconi anemia (FA) at age 6 years. The diagnosis was prompted by a history of frequent infections, easy bruising, recurrent epistaxis, and phenotype anomalies: short stature, microcephaly, microphthalmia, left renal agenesis with multicystic dysplasia of the right kidney, and an accessory nail phalanx of the right thumb (Figure 1a).

Initial evaluation revealed pancytopenia in peripheral blood, and a diepoxybutane (DEB) test confirmed chromosomal instability (198 aberrations in 53 metaphases; aberration index 3.7). Next-generation sequencing (NGS) was performed using a panel of cancer-associated genes to verify the diagnosis of FA. A variant of the nucleotide sequence *FANCA*(NM_000135.4):c.523-1G>C at the splicing site of exon 6 of the *FANCA* gene (chr16:89808368, rs1477653630) was identified. Segregation analysis detected a similar variant in the *FANCA* gene in a homozygous state in the middle 11-year-old sister (stature—134 cm; microcephaly—48 cm; and positive DEB test, without developmental abnormalities or cancer), whereas the patient’s father and mother were heterozygous carriers (Supplementary S1 and S2). According to the criteria of the ACMG (American College of Medical Genetics), this variant was classified as likely pathogenic (PM2, PM3, PVS1).

At the age of 10, the patient underwent an allogeneic hematopoietic stem cell (HSC) transplant from the bone marrow of an HLA-matched sibling (the eldest sister) due to progressive aplastic anemia requiring blood transfusions. The conditioning treatment included busulfan 4 mg/kg, thymoglobulin 10 mg/kg and fludarabine 150 mg/m^2^ on the background of standard concomitant therapy (diazepam). Also, he received antimycotic (voriconazole 400 mg/day) and antibacterial therapy (levofloxacin 500 mg/day, co-trimoxazole (sulfamethoxazole 400 mg + trimethoprim 80 mg) course for three days, rifaximin 200 mg/day), as well as hepatoprotector (ursodeoxycholic acid) 500 mg/day. For GVHD prophylaxis, rituximab (300 mg on day −1), methotrexate (5 mg/m^2^ on days +1, +3, +6, and +22), and cyclosporine A 20 mg starting on day −1 with monitoring of blood concentration were prescribed.

The post-transplant course was complicated by GVHD starting on day +23 involving the skin and lungs, which was managed with abatacept (250 mg intravenously over 1 h on day +24) and local treatment (tacrolimus and hydrocortisone twice daily). Acute GVHD resolved by day +39. Tacrolimus (0.25 mg twice daily, maintaining a serum concentration of 6–12 ng/mL) was administered to treat chronic GVHD, resulting in insufficient effect. After 18 months he was prescribed rituximab (375 mg/m^2^ over 6 h once a week for a course of 4 weeks) without significant clinical improvement. Following a multidisciplinary consultation ruxolitinib (0.3 mg/kg daily) and tacrolimus (0.25 mg twice daily, maintaining a serum concentration of 6–12 ng/mL) were prescribed for four years to treat chronic GVHD, resulting in partial control of the condition (Figure 1b–d). The reason for incomplete efficacy may be that the patient did not always follow the recommendations for hospitalization for control of serum concentration.

### 2.2. Presentation of SCC

**Figure 2:**
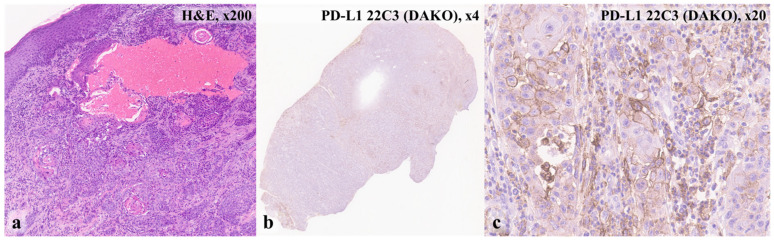
Histological examination (hematoxylin and eosin staining, ×200)—highly differentiated, keratinizing SCC (**a**); immunohistochemical investigation (PD-L1 22C3 (DAKO))—membrane staining of tumor cells is determined (**b**), as well as membrane-cytoplasmic staining of immunocompetent cells of varying intensity (**c**), CPS ≥ 1.

At age 15 years, the patient developed painless nodules on the eyelids, which slowly progressed over one year. He only sought medical attention once the lesions had begun to impair his vision (the patient’s visual acuity before SCC was OU—1.0). Initial surgical excision of a mass on the left upper eyelid was performed. During histological examination, foci of actinic keratosis, proliferative SCC in situ, SCC in situ with the onset of invasive growth, highly differentiated keratinizing SCC with infiltration of the entire dermis (Figure 2a), and keratoacanthoma were detected in various areas of the skin. Histological diagnosis was SCC, grade G1. Than immunohistochemistry was performed (Figure 2b,c)—PD-L1 positive status was revealed, combined positive score (CPS) ≥1.

Upon referral to our center at age 16 years, skin examination showed generalized hyperpigmentation, stigmata of chronic cutaneous GVHD, and, most strikingly, multinodular, exophytic, ulcerated tumors involving the upper and lower eyelids of both eyes, completely obscuring the palpebral fissures (Figure 1d). Additional tumor nodules were present on the right nasolabial fold, forehead, and left cheek.

### 2.3. Diagnostic Assessment

**Figure 3:**
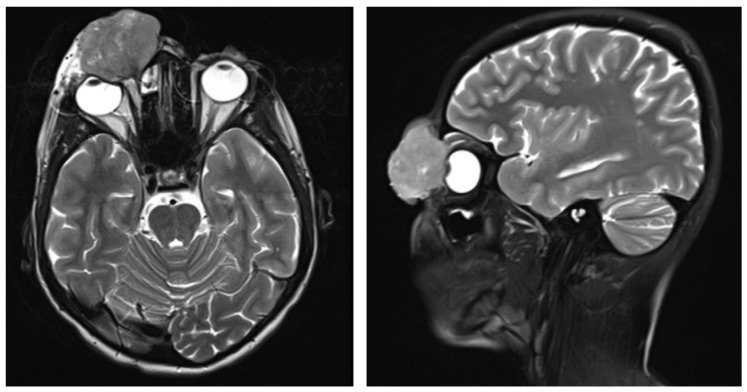
MRI of the orbits and brain (September 2024, 16 y.o.)—multinodular tumors, compressed eyeball and bones destruction.

Magnetic resonance imaging (MRI) of the orbits and brain (Figure 3) demonstrated a confluent mass measuring 3.7 × 4.1 × 4.9 cm involving the right eyelids, with extension to the nasal ala and dorsum. The tumor invaded and destroyed the nasal bone, anterior ethmoid cells, frontal bone, and frontal process of the maxilla, with infiltration into the anteromedial right orbit. Separate nodules were noted in the right lower lip and left eyelids. The disease was staged as locally advanced cutaneous SCC, T3N0M0, Stage III.

Immunophenotyping of peripheral blood to assess secondary immunodeficiency revealed no significant changes in the composition of lymphocyte subpopulations.

### 2.4. Treatment and Clinical Course

**Figure 4:**
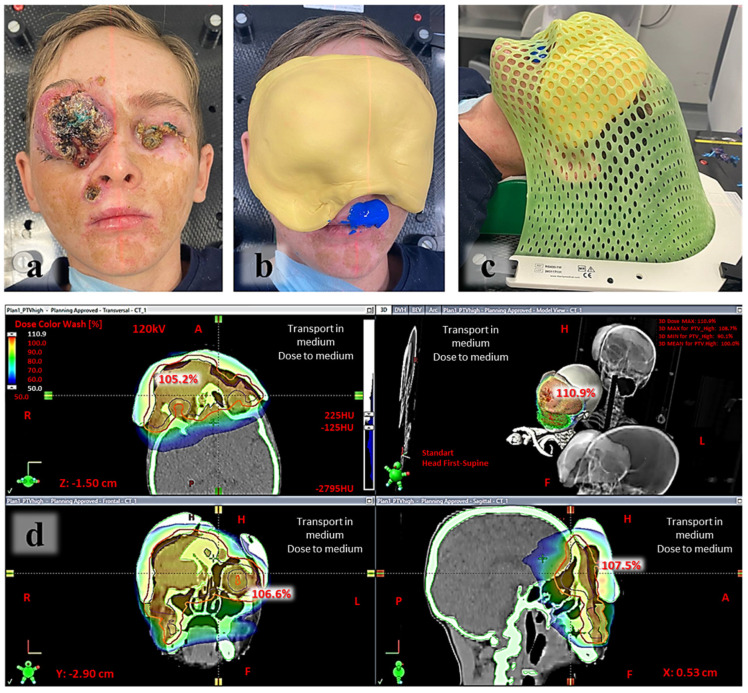
General appearance of the patient at the time of preparation for radiotherapy (**a**); pre-radiation topometry with individualized bolus fabrication (**b**,**c**); radiotherapy plans up to 56 Gy (**d**).

Due to the extent of the disease, radical surgical resection was not feasible. The patient underwent debulking of the left upper eyelid tumor and incisional biopsy of the right upper eyelid mass. Subsequently, he received intensity-modulated radiotherapy (IMRT) to all visible tumor sites (Figure 4).

Due to significant tumor bleeding, the first five fractions of radiotherapy were delivered at a single dose of 1.5 Gy per session. Starting from the sixth fraction, treatment continued with a single dose of 2 Gy per session. Upon reaching a total dose of 56 Gy, repeat topometric assessment and treatment plan recalculation were performed due to a positive response. The tumor regression (reduction in the tumor in the right orbital region by 63.25%) was significant but still insufficient for further surgical resection.

This case was discussed on a tumor board that included hematologists, transplant physicians, oncologists, clinical pharmacologists, and ethics committee members. Immunotherapy with pembrolizumab was prescribed as off-label treatment (compassionate use), because other treatment options had been exhausted. It was approved by the Ethics Committee of the N.N. Blokhin National Medical Research Center of Oncology. The immunotherapy was performed in a high-volume expert center with experience in managing post-transplant complications and immune-related adverse events provided the necessary infrastructure to attempt this approach. The patient was fully informed about the potential risks of immune checkpoint inhibitors after allo-HSCT, including severe GVHD, immune encephalitis, and death. Written informed consent was obtained.

**Figure 5:**
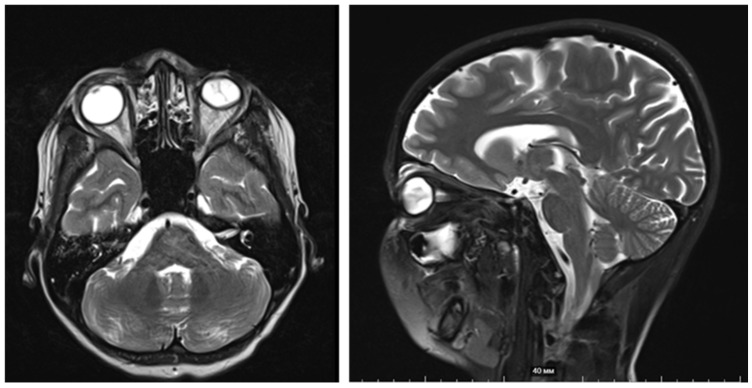
MRI of the orbits and brain (March 2025, 16 y.o.) after radiotherapy and 3 months of immunotherapy (5 infusions): decreased soft-tissue tumor mass.

The patient started pembrolizumab at a standard dose of 200 mg intravenously every three weeks just after completing radiotherapy. An initial MRI at 3 months showed a favorable response, with regression of the frontal lesion (now 1.8 × 2.0 × 2.0 cm) and no further orbital invasion (Figure 5). This may be the result of a combination of immunotherapy and previous radiation therapy.

**Figure 6:**
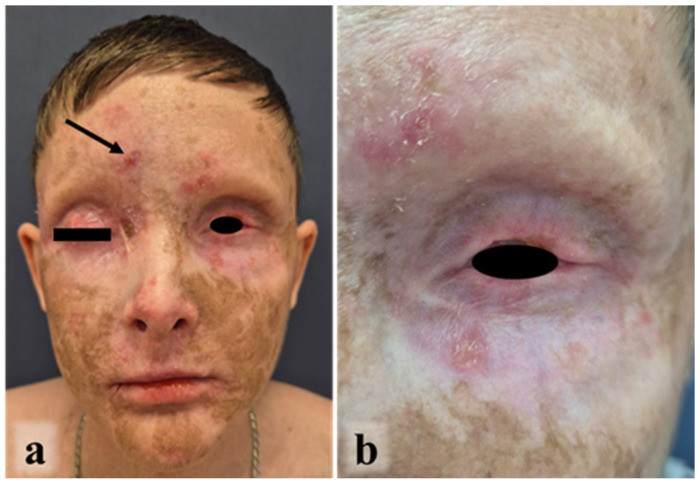
Examination of the patient (July 2025, 16 y.o.): residual tumor after treatment, indicated by an arrow (**a**); post-radiation atrophy of the eyelids (**b**).

No severe immune-related adverse events were reported during the first 7 months of continuous pembrolizumab treatment. Nevertheless, a punch biopsy revealed the persistence of viable SCC in a residual facial lesion (Figure 6a), and post-radiation atrophy of the eyelids was also identified (Figure 6b).

### 2.5. Disease Progression and Toxicity

**Figure 7:**
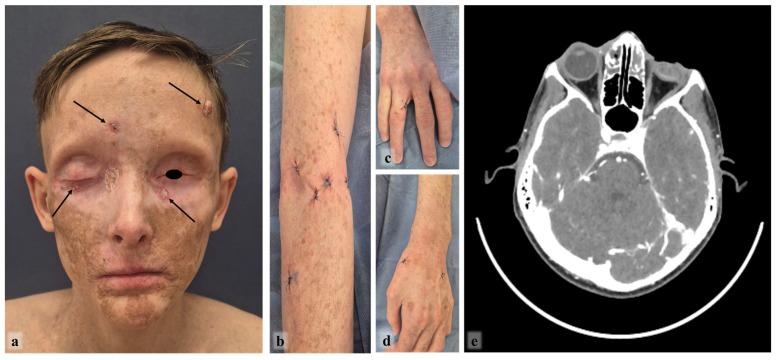
Examination of the patient (February 2026, 17 y.o.): growth of residual tumor and new foci of SCC (indicated by arrows) of the facial skin (**a**); surgically removed foci of SCC of the skin in the upper limbs (**b**–**d**); computed tomography—additional tissues in the frontal area and uneven accumulation of contrast (**e**).

After a total of 14 months of pembrolizumab treatment (at age 17 years), the patient showed disease progression. The residual forehead lesion had enlarged, and examination revealed nine new, histologically confirmed SCC nodules on the lower eyelids, left temporal area, and upper limbs (Figure 7a–d). Concurrently, the patient developed severe, progressive malnutrition. His weight decreased from an already low baseline of 52 kg to 36 kg (a 30% loss), resulting in a body mass index consistent with severe cachexia. He reported anorexia and fatigue but no overt diarrhea or vomiting, suggesting a possible immune-related anorexia/cachexia syndrome rather than enterocolitis. Computed tomography confirmed local progression with indistinct soft tissue infiltration in the frontal region (Figure 7e).

**Figure 8:**
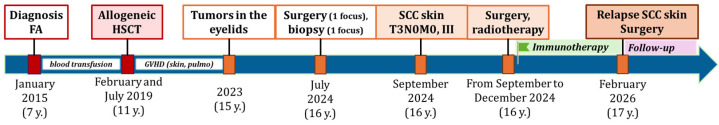
The stages of the patient’s treatment.

Given the clear evidence of both disease progression and life-threatening toxicity, the oncology multidisciplinary team recommended discontinuation of pembrolizumab. The patient subsequently underwent surgical excision of the nine new cutaneous lesions, all confirmed as SCC. A timeline of the patient’s treatment journey is summarized in Figure 8.

### 2.6. Follow-Up and Family Management

The patient is currently alive with this disease and is under close medical surveillance. Recently, he underwent first stage of reconstructive surgery at an ophthalmology clinic and now he can distinguish between light and shadow. His sister, who is homozygous for the *FANCA* mutation, is enrolled in a high-risk cancer surveillance program that includes regular dermatological and otolaryngological examinations. At this time, neither of them is receiving any additional treatment.

## 3. Discussion

### 3.1. Standard Management of Cutaneous SCC According to NCCN Guidelines

Treatment options in the NCCN Guidelines for Squamous Cell Skin Cancer are based on risk stratification [13]. For cutaneous SCC (cSCC) in situ electrodesiccation, curettage, topical agents (5-fluorouracil, calcipotriene, imiquimod, diclofenac, and tazarotene), photodynamic therapy and cryotherapy can be prescribed [14]. Surgical excision with 4 to 6 mm clinical margins is the gold standard for the treatment of low-risk cSCC, whereas Mohs micrographic surgery (MMS) was recommended for high-risk cSCC. Moreover, MMS is emerging as the preferred technique for the resection of cSCC located in the face (aesthetically challenging anatomical areas) [13].

Nonsurgical approaches may be considered for high-risk cSCC or in special situations in which surgery is not feasible. Primary or adjuvant radiation therapy is an effective treatment option for this group of patients. Also, for the treatment of metastatic cSCC, targeted therapy (epidermal growth factor inhibitors) and chemotherapy (cisplatin), as a single agent or in combination (chemoradiation therapy), may be considered.

Since 2018 (cemiplimab) and 2020 (nivolumab and pembrolizumab) immunotherapy were approved by FDA (Food and Drug Administration) for advanced cSCC [15,16]. Cemiplimab has demonstrated clear benefit in both neoadjuvant and adjuvant settings for patients with high-risk SCC following surgery and radiation therapy. Also, anti-PD-1/PD-L1 immunotherapy showed a good response for treating locally advanced cutaneous SCC of the periocular region [17].

However, it is important to remember the adverse effects seen in the patients treated with FDA-approved PD-1/PD-L1-antibodies—diarrhea, fatigue, nausea, pruritus, rash, reduced appetite, constipation, and life-threatening complications, like immune-mediated encephalitis, myocarditis, pneumonitis, renal and hepatic disorder, myasthenia gravis and tumor pseudoprogression phenomenon [18]. According to FAERS (FDA Adverse Event Reporting System), male sex and older age (the mean age of patients who died was 66.3 years) were negative prognostic factors, associated with high risk of adverse effects after pembrolizumab treatment. GVHD is also a possible complication of immunotherapy. Haverkos BM et al., 2017 showed the rapid onset of severe and treatment-refractory GVHD in patients receiving immunotherapy for relapses after allo-HSCT, especially in the case with a history of GVHD [19].

Our patient did not have active GVHD at baseline and received careful monitoring, but this does not eliminate the risk. Immunotherapy should be used with extreme caution, especially for patients after allo-HSCT, and only in specialized centers with experience in managing ICI-related and transplant-related toxicities.

### 3.2. The Surgical Imperative and the Paradox of Chemoradiotherapy of SCC Treatment in Patients with FA

The management of locally advanced SCC in FA patients presents an agonizing therapeutic dilemma [20]. Most reports about therapeutic experience in this field are isolated cases or small series; there are no randomized clinical trials. Surgery remains the undisputed standard of care, and its prioritization in our patient achieved initial local control. However, in cases where radical resection is not possible, such as in this instance, with extensive involvement of the orbital and facial bones, clinicians must consider DNA-damaging therapies, but with caution.

Platinum-containing chemotherapy is a common regimen for the treatment of sporadic SCC. But in patients with FA, it results in multiple DNA-crosslinks and excessive toxicity—cytopenia, high-grade mucositis, dysphagia, bleeding. Conventional chemotherapy was described just in single cases in the literature—carboplatin in monotherapy, a combination of cisplatin, bleomycin, and methotrexate or cisplatin with 5-ftoruracile, etc. [21]. More often patients receive chemoradiotherapy.

Radiotherapy remains the optimal option for SCC localized in the oral cavity, pharynx, larynx and anogenital region, albeit with careful dose modification and close monitoring for toxicity. While effective in achieving tumor regression (as seen in our patient at 56 Gy), radiotherapy is intrinsically carcinogenic in FA patients due to their underlying DNA repair defect [22]. Ionizing radiation creates double-strand breaks that FA cells cannot accurately repair, preferentially utilizing error-prone NHEJ (non-homologous joining of the ends) or MMEJ (micro-homologous joining of the ends) pathways. This mechanism is not directly responsible for acute toxicity, but it causes genomic instability which may contribute to the development of secondary malignant tumors in the radiation field.

The pattern of recurrence in our patient—with the emergence of new cSCC foci both within and outside the irradiated area—clearly illustrates the effect of radiation against the background of GVHD. The in-field progression may represent radiation-induced carcinogenesis, while out-of-field metastases reflect the inherent aggressiveness of cSCC in the setting of FA and chronic GVHD-induced immunosuppression. This observation supports the growing consensus that radiotherapy in FA should be limited to carefully selected cases where surgical options have been exhausted, and even in such cases meticulous planning is required to minimize radiation exposure.

### 3.3. Immunotherapy: A Controversial Option in Fanconi Anemia

Immune checkpoint inhibitors (ICIs) have transformed oncology, including the treatment of cSCC in immunocompetent patients [23]. Some authors have suggested using ICIs as monotherapy in the first line of unresectable SCC [24] or in cases of perineural invasion [25]. Recent studies have shown that low doses of radiotherapy (<3 Gy per fraction) improve the sensitivity of primary and metastatic lesions to ICIs [26]. Koukourakis IM et al., 2025 demonstrated a highly positive effect of using a therapeutic algorithm involving upfront cemiplimab, followed by hypofractionated radiation therapy in patients with locally advanced SCC [27]. The C-POST clinical trial also confirmed the efficacy of using ICIs in the treatment of locally advanced cSCC in adult patients who had previously received surgery and radiation therapy [28]. Thus, the use of this combination could potentially be an optimal option for the treatment of unresectable cSCC.

However, their application in FA rests on an unstable theoretical foundation. The rationale for ICI use in FA derives from the high mutational burden of FA-associated tumors—a consequence of genomic instability that should, in theory, generate abundant neoantigens and render tumors “hot” and responsive to PD-1 blockade [29]. Our case and the limited published experience (as shown in Table 1) with fatal complications suggest that this theoretical benefit is offset by substantial risks [21,30,31].

For the five patients whose condition could be evaluated (including our own), the results were extremely disappointing. Three of the five patients have died, with survival ranging from 10.5 to 77 months. Objective responses, when observed, were transient. Our patient achieved initial disease control but experienced multifocal progression after 14 months of continuous pembrolizumab—a pattern consistent with acquired resistance.

The toxicity profile is even more concerning. While FA patients are assumed to be at lower risk for immune-related adverse events due to their impaired immune systems, this assumption may be dangerously flawed. According to the literature, one patient died from encephalitis and aspiration pneumonia, both of which were presumed to be caused by nivolumab [21].

Despite normal laboratory parameters, our patient developed severe, progressive cachexia (30% weight loss) without gastrointestinal symptoms, suggesting a possible immune-mediated anorexia/metabolic syndrome that required treatment discontinuation.

The limited efficacy and significant toxicity observed in our patient and in the reported cases suggest that ICIs are not a reliable salvage strategy for advanced SCC in this population. If ICIs are considered, they should be used only in specialized centers with expertise in both FA and immunotherapy, along with intensive monitoring for atypical toxicities including nutritional decline. Future research must prioritize the identification of predictive biomarkers for both response and toxicity in FA patients. Comprehensive genomic profiling of FA-associated SCC, characterization of the tumor immune microenvironment, and prospective collection of toxicity data through international registries are urgently needed. Until such data emerge, the treatment of advanced SCC in FA will remain a formidable clinical challenge requiring individualized, multidisciplinary decision-making.

## 4. Conclusions

The potential risk of developing aggressive solid tumors following HSCT underscores the need for lifelong, rigorous dermatologic and otorhinolaryngologic surveillance of all patients with FA, particularly those with additional risk factors such as a history of HSCT, GVHD, and sun exposure.

Here, we have reported the first case of immunotherapy for skin SCC in a pediatric patient with FA. Our experience confirms the central role of surgical treatment as the cornerstone of therapy for SCC in FA. Although the use of pembrolizumab might have produced some transient therapeutic effects, the severe toxicities observed in this (multifocal progression and the development of life-threatening cachexia) and other reported cases do not support its routine use outside the setting of a clinical study. Thus, the risk–benefit profile of PD-1 blockade in this patient group is far less favorable than suggested by theoretical models predicting high efficacy.

## 5. Limitations

Since this is a case report, our conclusions are inherently limited in generalizability. A notable limitation is the lack of data on tumor mutational burden (TMB)—marker that could predict response to therapy with immune checkpoint inhibitors (ICIs). However, obtaining a sufficient amount of tumor tissue for such analyses in a patient with multifocal disease who had undergone intensive therapy proved to be a difficult task. Furthermore, the combined effects of prior radiation therapy and chronic GVHD make it difficult to attribute the observed disease progression and toxicity solely to pembrolizumab. Nevertheless, the temporal association between the start of ICI treatment and the described events is compelling.

## Figures and Tables

**Table 1:** Global experience in the use of immunotherapy in patients with FA.

Source	Gender, Age of SCC, Gene	Location and Stage of SCC	HSCT (Age)	Treatment of Primary Tumor	Relapse or Progression	Treatment of Recurrent Tumor	Outcome, SD (Months)
Beckham TH et al., 2019 [21]	M 33 y.o. *FANCC*	Larynx pT3N2c	Yes (17 y.o.)	Surgery, CT, RT	Relapse after 3 months	RT, IT (N)	Dead, SD—12
Beckham TH et al., 2019 [21]	F 31–35 y.o. *FANCA*	Tongue (3 foci) pT1N0	No	Surgery	Relapse after 1 year	Surgery, RT, PCT, IT (T, D)	Dead, SD—77
Lewis LM et al., 2020 [30]	F 27 y.o. *FANCG*	Left buccal mucosa cT1N0M0	No	Surgery	Relapse after 8 months	Surgery, RT, IT (P)	Alive
Nagano K et al., 2022 [31]	F 18 y.o. ND	Tongue cT4bN2cM1	Yes (18 y.o.)	IT (P)	Progression after 3.3 months	CT, IT (N)	Dead, SD—10.5
Our case, 2026	M 16 y.o. *FANCA*	Skin of the face T3N0M0	Yes (11 y.o.)	Surgery, RT, IT (P)	Relapse after 7 months	Surgery, IT (P)	Alive, second relapse

Abbreviations: CT—chemotherapy (cetuximab in all cases); D—durvalumab; HSCT—hematopoietic stem cell transplantation; IT—immunotherapy; N—nivolumab; ND—no data; P—pembrolizumab; PCT—polychemotherapy (cetuximab plus paclitaxel); RT—radiotherapy; SCC—squamous cell carcinoma; SD—survivalduration; T—tremelimumab.

## Data Availability

The original contributions presented in the study are included in the article/Supplementary Material. Further inquiries can be directed to the corresponding author.

## Supplementary Materials

The following supporting information can be downloaded at: https://www.mdpi.com/article/10.3390/ijms27104366/s1.

## Abbreviations

The following abbreviations are used in this manuscript:

ACMG	American College of Medical Genetics and Genomics
CPS	Combined positive score
cSCC	Cutaneous squamous cell carcinoma
DEB	Diepoxybutane
DNA	Deoxyribonucleic acid
FA	Fanconi anemia
FAERS	FDA Adverse Event Reporting System
FDA	Food and Drug Administration
GVHD	Graft-versus-host disease
HLA	Human leukocyte antigen
HSCT	Hematopoietic stem cell transplantation
IT	Immunotherapy
MMEJ	Micro-homologous joining of the ends
MMS	Mohs micrographic surgery
MRI	Magnetic resonance imaging
NGS	Next-generation sequencing
NHEJ	Non-homologous joining of the ends
PM	Pathogenic moderate
PVS	Pathogenic very strong
SCC	Squamous cell carcinoma

## References

[1] Woodward E.R., Meyer S. (2021). Fanconi Anaemia, Childhood Cancer and the BRCA Genes. Genes.

[2] Fanconi G. (1927). Familial infantile pernicious-like anemia (pernicious blood picture and constitution). Jahrb. Für Kinderheilkd..

[3] García-de-Teresa B., Rodríguez A., Frias S. (2020). Chromosome Instability in Fanconi Anemia: From Breaks to Phenotypic Consequences. Genes.

[4] Wang L.C., Gautier J. (2010). The Fanconi anemia pathway and ICL repair: Implications for cancer therapy. Crit. Rev. Biochem. Mol. Biol..

[5] Shimamura A., Montes de Oca R., Svenson J.L., Haining N., Moreau L.A., Nathan D.G., D’Andrea A.D. (2002). A novel diagnostic screen for defects in the Fanconi anemia pathway. Blood.

[6] Kutler D.I., Singh B., Satagopan J., Batish S.D., Berwick M., Giampietro P.F., Hanenberg H., Auerbach A.D. (2003). A 20-year perspective on the International Fanconi Anemia Registry (IFAR). Blood.

[7] Lum S.H., Eikema D.J., Piepenbroek B., Wynn R.F., Samarasinghe S., Dalissier A., Kalwak K., Ayas M., Hamladji R.M., Yesilipek A. (2024). Outcomes of hematopoietic stem cell transplantation in 813 pediatric patients with Fanconi anemia. Blood.

[8] Svojgr K., Sumerauer D., Puchmajerova A., Vicha A., Hrusak O., Michalova K., Malis J., Smisek P., Kyncl M., Novotna D. (2016). Fanconi anemia with biallelic FANCD1/BRCA2 mutations—Case report of a family with three affected children. Eur. J. Med. Genet..

[9] Choi J., Jung M. (2025). Head and Neck Cancer in Fanconi Anemia: Clinical Challenges and Molecular Insights into a DNA Repair Disorder. Cancers.

[10] Reinhard H., Peters I., Gottschling S., Ebell W., Graf N. (2007). Squamous cell carcinoma of the tongue in a 13-year-old girl with Fanconi anemia. J. Pediatr. Hematol. Oncol..

[11] Anak S., Yalman N., Bilgen H., Sepet E., Deviren A., Gürtekin B., Tunca F., Başaran B. (2020). Squamous cell carcinoma development in Fanconi anemia patients who underwent hematopoietic stem cell transplantation. Pediatr. Transplant..

[12] Furquim C.P., Pivovar A., Amenábar J.M., Bonfim C., Torres-Pereira C.C. (2018). Oral cancer in Fanconi anemia: Review of 121 cases. Crit. Rev. Oncol. Hematol..

[13] Kim J.Y.S., Kozlow J.H., Mittal B., Moyer J., Olenecki T., Rodgers P., Work Group, Invited Reviewers (2018). Guidelines of care for the management of cutaneous squamous cell carcinoma. J. Am. Acad. Dermatol..

[14] Beach S.C., Cusick A.S., Farberg A.S., Trotter S.C. (2025). A Comprehensive Narrative Review of the Challenges Surrounding Cutaneous SCC. Dermatol. Ther..

[15] Ahmed S.R., Petersen E., Patel R., Migden M.R. (2019). Cemiplimab-rwlc as first and only treatment for advanced cutaneous squamous cell carcinoma. Expert Rev. Clin. Pharmacol..

[16] Chedid M.F., Tregnago A.C., Riva F., Prediger L., Agarwal A., Mattei J. (2025). Indications and Mechanisms of Action of the Main Treatment Modalities for Non-Melanoma Skin Cancer. Life.

[17] Bineshfar N., Meller L., Rong A.J., Johnson T.E., Lee W.W. (2026). Immune Checkpoint Inhibitors for the Treatment of Periorbital Non-Melanoma Skin Cancers. Semin. Ophthalmol..

[18] Xu H., Huang Y., Zhao N., Hu H., Cao D. (2025). Retrospective analysis of pembrolizumab-related adverse reactions and death outcomes based on the FAERS database. BMC Cancer.

[19] Haverkos B.M., Abbott D., Hamadani M., Armand P., Flowers M.E., Merryman R., Kamdar M., Kanate A.S., Saad A., Mehta A. (2017). PD-1 blockade for relapsed lymphoma post-allogeneic hematopoietic cell transplant: High response rate but frequent GVHD. Blood.

[20] Chaithanya D., Lakshmi J.S., Christopher J., Satish Srinivas K. (2025). A Deadly Duet: Fanconi Anemia (FA) With Head and Neck Cancer. Cureus.

[21] Beckham T.H., Leeman J., Jillian Tsai C., Riaz N., Sherman E., Singh B., Lee N., McBride S., Higginson D.S. (2019). Treatment modalities and outcomes of Fanconi anemia patients with head and neck squamous cell carcinoma: Series of 9 cases and review of the literature. Head Neck.

[22] Deng X., Tchieu J., Higginson D.S., Hsu K.S., Feldman R., Studer L., Shaham S., Powell S.N., Fuks Z., Kolesnick R. (2021). Disabling the Fanconi Anemia Pathway in Stem Cells Leads to Radioresistance and Genomic Instability. Cancer Res..

[23] Shalhout S.Z., Emerick K.S., Kaufman H.L., Miller D.M. (2021). Immunotherapy for Non-melanoma Skin Cancer. Curr. Oncol. Rep..

[24] Maubec E., Boubaya M., Petrow P., Beylot-Barry M., Basset-Seguin N., Deschamps L., Grob J.J., Dréno B., Scheer-Senyarich I., Bloch-Queyrat C. (2020). Phase II Study of Pembrolizumab As First-Line, Single-Drug Therapy for Patients With Unresectable Cutaneous Squamous Cell Carcinomas. J. Clin. Oncol..

[25] Morecroft R.A., Phillipps J.S., Gou L., Bhatt A.A., Kim S., Mohammadi H., Dronca R.S., Doonan B., Chen R., Zhao Y. (2025). Immunotherapy and Radiation for Clinical Perineural Invasion in Cutaneous Squamous Cell Carcinoma. Cancers.

[26] Zhu S., Wang Y., Tang J., Cao M. (2022). Radiotherapy induced immunogenic cell death by remodeling tumor immune microenvironment. Front. Immunol..

[27] Koukourakis I.M., Karpouzis A., Filippatos K., Mamalis P., Kakagia D., Giatromanolaki A., Kouloulias V., Zygogianni A., Koukourakis M.I. (2025). Integration of immunotherapy and radiotherapy in a therapeutic algorithm for locally advanced squamous cell skin cancer. Med. Oncol..

[28] Cavalieri S., Ottini A., Bergamini C., Alfieri S., Nuzzolese I., Colombo E., Lombardi Stocchetti B., Licitra L. (2025). Adjuvant anti-PD-1 therapy in high-risk cutaneous squamous-cell carcinoma: Post hoc insights from the C-POST and KEYNOTE-630 studies. Immunooncol. Technol..

[29] Migden M.R., Rischin D., Schmults C.D., Guminski A., Hauschild A., Lewis K.D., Chung C.H., Hernandez-Aya L., Lim A.M., Chang A.L.S. (2018). PD-1 Blockade with Cemiplimab in Advanced Cutaneous Squamous-Cell Carcinoma. N. Engl. J. Med..

[30] Lewis L.M., Tang A.L., Wise-Draper T.M., Myers K.C., Greenberger J.S., Takiar V. (2020). Successful use of a therapeutic trial of graduated volume and dose escalation for postoperative head and neck radiotherapy in a Fanconi anemia patient. Head Neck.

[31] Nagano K., Osaki M., Hatanaka A., Kinoshita S. (2022). Pembrolizumab for Fanconi anemia with advanced tongue cancer. Otolaryngol. Case Rep..

