# Supplementary material for: Squamous Cell Carcinoma of the Skin in a Teenager with Fanconi Anemia: A Challenging Treatment
Source: Int J Mol Sci. 2026 May 14;27(10):4366. doi: 10.3390/ijms27104366 (PMC13207001; doi:10.3390/ijms27104366)
Supplement: Supplementary file 1 [file ijms-27-04366-s001.zip › Supplementary S2.pdf]

Supplementary S2. Materials and Methods

Genomic DNA was isolated from patient's skin without lesions using the QIAmp DNA Mini Kit (Qiagen, Hilden, Germany), then NGS sequencing was performed. Library was prepared with KAPA HyperPrep Kit (Roche, Basel, Switzerland) and hybridized with coding regions of 198 genes. Sample was sequenced on MiSeq (Illumina, San Diego, CA, USA). Technical characteristics were paired-end sequencing, 300 cycles, and 250–300× coverage depth. Sequencing data were processed and aligned to the reference genome sequence GRCh (hg38).

Interpretation of the identified variant was carried out according to the ACMG guidelines (doi: 10.1038/gim.2015.30) using ClinVar (https://www.ncbi.nlm.nih.gov/clinvar, accessed on 25 November 2025), and Franklin Genoox (https://franklin.genoox.com, accessed on 25 November 2025) databases.

For patient's relatives DNA was isolated from blood leukocytes and Sanger sequencing was performed. Primers pair:

Forward: AAACCCGTCTGATTCTGGGCT

Reverse: CAAGTGTTGGGCAACGGTGT
