# Supplementary figures and images for: Squamous Cell Carcinoma of the Skin in a Teenager with Fanconi Anemia: A Challenging Treatment
Source: Int J Mol Sci. 2026 May 14;27(10):4366. doi: 10.3390/ijms27104366 (PMC13207001; doi:10.3390/ijms27104366)

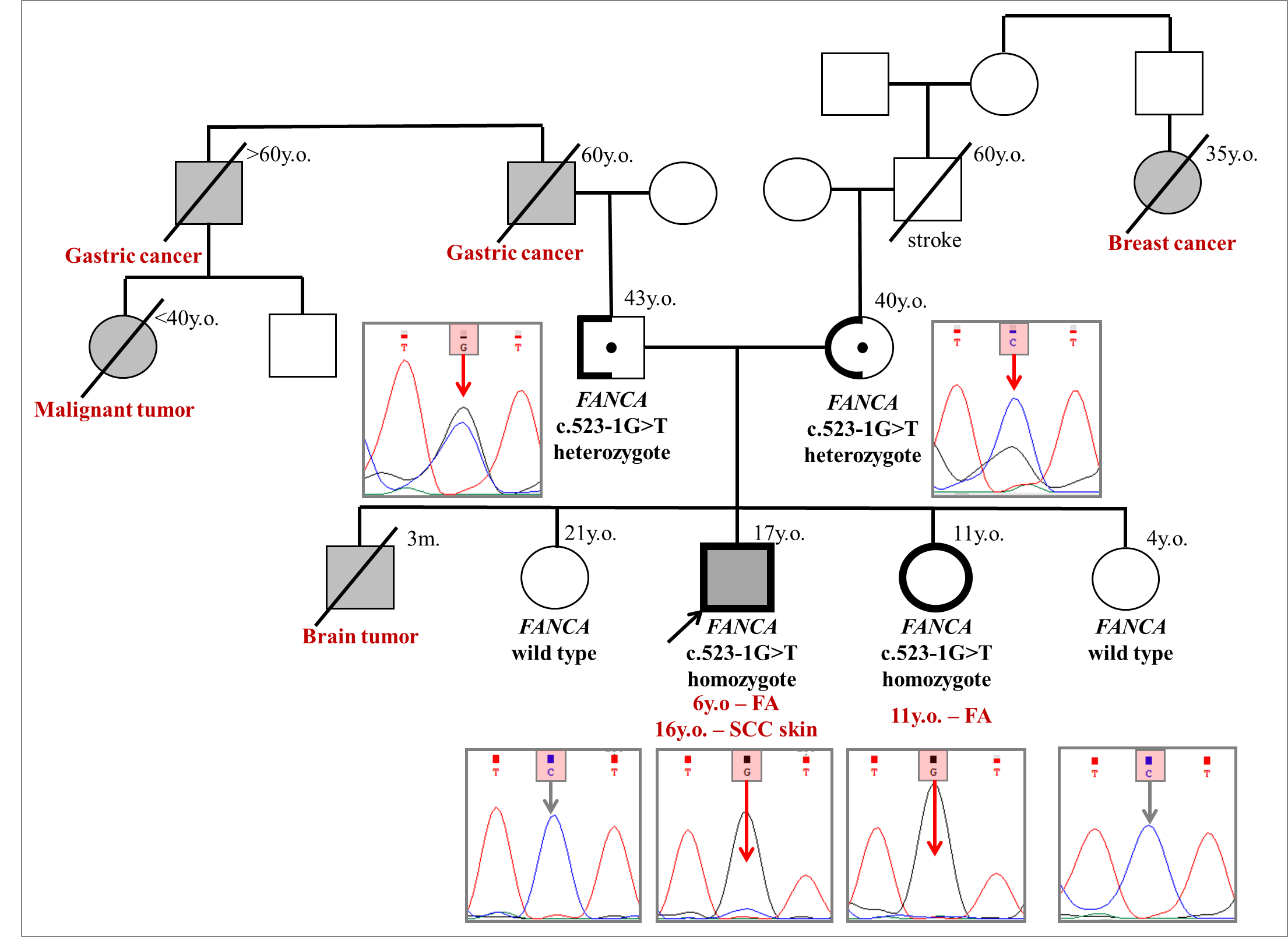

Supplement: Supplementary file 1 [file ijms-27-04366-s001.zip › Supplementary S1. Pedigree and genetic analysis.tif]
